# Insomnia and urban neighbourhood contexts – are associations modified by individual social characteristics and change of residence? Results from a population-based study using residential histories

**DOI:** 10.1186/1471-2458-12-810

**Published:** 2012-09-20

**Authors:** Natalie Riedel, Kateryna Fuks, Barbara Hoffmann, Simone Weyers, Johannes Siegrist, Raimund Erbel, Anja Viehmann, Andreas Stang, Joachim Scheiner, Nico Dragano

**Affiliations:** 1 Faculty of Spatial Planning, Institute of Spatial Planning, TU Dortmund University, Dortmund, Germany; 2 IUF- Leibniz Research Institute for Environmental Medicine, Düsseldorf, Germany, and Heinrich-Heine University of Düsseldorf, Medical Faculty, Düsseldorf, Germany; 3 Institute of Medical Sociology, Heinrich Heine University of Düsseldorf, Düsseldorf, Germany; 4 West German Heart Centre, University of Duisburg-Essen, University Hospital Essen, Essen, Germany; 5 Institute of Medical Informatics, Biometry and Epidemiology, University of Duisburg-Essen, University Hospital Essen, Essen, Germany; 6 Institute of Clinical Epidemiology, Martin-Luther-University of Halle-Wittenberg, Halle, Germany; 7 Faculty of Spatial Planning, Department of Transport Planning, TU Dortmund University, Dortmund, Germany

**Keywords:** Insomnia, Neighbourhood unemployment, Residential turnover, Income, Education, Social isolation, Change of residence.

## Abstract

**Background:**

Until now, insomnia has not been much of interest in epidemiological neighbourhood studies, although literature provides evidence enough for insomnia-related mechanisms being potentially dependent on neighbourhood contexts. Besides, studies have shown differences in sleep along individual social characteristics that might render residents more vulnerable to neighbourhood contextual exposures. Given the role of exposure duration and changes in the relationship between neighbourhoods and health, we studied associations of neighbourhood unemployment and months under residential turnover with insomnia by covering ten years of residential history of nearly 3,000 urban residents in the Ruhr Area, Germany.

**Methods:**

Individual data were retrieved from the Heinz Nixdorf Recall Study, a population-based study of randomly chosen participants from adjacent cities, which contains self-rated insomnia symptoms and individual social characteristics. Participants’ residential addresses were retrospectively assessed using public registries. We built individually derived exposure measures informing about mean neighbourhood unemployment rates and months under high residential turnover. These measures were major predictors in multivariate logistic regressions modelling the association between social neighbourhood characteristics and insomnia in the whole sample and subgroups defined by low income, low education, social isolation, and change of residence. Traffic-related noise, age, gender, economic activity, and education were considered as covariates.

**Results:**

Nearly 12 per cent of the participants complained about insomnia. Associations of neighbourhood unemployment with insomnia were more consistent than those of residential turnover in the whole sample (adjusted OR 1.42, 95% CI 1.00-2.03 for neighbourhood unemployment and OR 1.33, 95% CI 0.78-2.25 for residential turnover in the highest exposure categories). In low-income and socially isolated participants, neighbourhood unemployment odds of reporting insomnia were particularly elevated (adjusted OR 2.90, 95% CI 1.39-6.02 and OR 3.32, 95% CI 1.11-9.96, respectively). Less educated participants displayed relatively high odds of reporting insomnia throughout all upper neighbourhood unemployment exposure categories. Change of residence weakened associations, whereas undisrupted exposure sharpened them by trend.

**Conclusions:**

Our findings hint at multiple stressors being effective in both the neighbourhood context and individual resident, possibly reflecting precarious life situations undermining residents’ sleep and health chances. Moreover, our results suggest a temporal dependency in the association between neighbourhood and insomnia.

## Background

Urban health research in social epidemiology suggests that social neighbourhood characteristics are involved in urban residents’ developing chronic diseases. A growing body of evidence supports causal pathways associating contextual features with a variety of outcomes such as cardiovascular and -metabolic diseases, depression or unhealthy lifestyles [1]. In spite of its high prevalence in the general population [2,3], insomnia has not been much of interest in epidemiological neighbourhood studies [4]. Only a few studies have explored whether social neighbourhood characteristics might be associated with sleep problems [5,6].

Further exploration of this subject is desirable, since poor sleep affects major physiological and psychological functions, particularly in the elderly [7-9]. Insomnia is assumed to play a crucial role in the complex relationship between stress and health [10]. By undermining regulatory systems and immune functions in its wake, chronic insomnia can aggravate other acute and chronic diseases, which, in turn, may impair sleep quality. Among others, interrelations for subclinical and clinical conditions are known for inflammation, hormonal dysfunction, cancer, chronic pain, cardiovascular and cerebrovascular disease, neurological disease, diabetes, obesity, depression, and negative affectivity [7-9,11].

We conceive macro-social inequalities to be the cause of socio-spatially unequal quantities and qualities of contextual stressors and resources, and population compositions in the urban realm [12-14]. In this perspective, the urban community and neighbourhood emerge as an intermediate setting where the relation between both contextual and individual stressors and resources may pre-determine community and individual health. Adopting such a ‘multiple stressor approach’ [15,16] and focussing on the interdependence of stress, sleep and health [10], we propose stress to be a plausible key factor linking social neighbourhood characteristics to insomnia.

In this explorative work, we studied two neighbourhood contextual characteristics reflecting an array of residential psychosocial and physical stressors which might trigger adverse behavioural, psychological and physiological changes and thereby induce insomnia: *neighbourhood unemployment* and *residential turnover*. Despite the sleep-related knowledge gap in the field of neighbourhood research, literature provides us with sufficient evidence to examine associations between these two neighbourhood exposures and the presence of insomnia.

*Neighbourhood unemployment* represents neighbourhood or area socioeconomic status [17] and is supposed to structure diverse sleep-related stressors across neighbourhoods, e.g. substandard housing conditions in terms of inadequate insulation, dilapidations, air pollution, and overcrowding, neighbourhood disorder as measured by perceived crime and safety, litter and lack of attractiveness, or proximity to noisy industrial sites and heavy traffic loads [5,6,18,19]. In a recent study on young adults in the US, a gender-, age- and race-adjusted association of neighbourhood socioeconomic status with insomnia symptoms was found [19]. However, effects were diminished when individual socioeconomic and -demographic, lifestyle, and physical and mental health variables were controlled for.

*Residential turnover* in urban neighbourhoods *–* as opposed to residential stability [20] – might be another structural determinant of insomnia, particularly, if we base our assumptions on stress-related pathways. To the best of our knowledge, there has been no study dealing with the effect of residential turnover on insomnia or sleep quality so far. But residential stability has been already identified as a factor attenuating stress in urban residents through social networks and resources [21]. The dynamic of residential stability and turnover might be a driver of contradictory social processes and interactions: either social cohesion as a protective resource or social isolation as a harmful psychosocial stressor. In face of neighbourhood unemployment and its associated intermediate stressors, for instance, residential stability might give rise to strong social bonds, delivering mutual support and compensating residents’ disadvantages in market regimes and welfare subsidies, housing and regeneration policies. In a study accounting for neighbourhood unemployment, residential stability was thought to promote chances for survival after myocardial infarction [22]. Recent findings on mortality and morbidity rates might further corroborate the hypothesis of residential stability being beneficial for health: As compared to areas characterised by a stable migration balance and a low turnover, mortality and morbidity rates were distinctively elevated in deprived areas with changes in the population due to decrease [23,24]. This phenomenon might be indicative of a downward spiral eroding health and health-related resources in the neighbourhood: When healthier residents migrate to wealthier neighbourhoods, taking social resources with them, psychosocial stress might grow among remaining residents. As a consequence, the impact of neighbourhood deprivation on health intensifies and socio-spatial inequalities in health enlarge. In contrast, residential stability combined with neighbourhood deprivation could also signal selective entrapment, causing psychological distress in residents not being able to move [20,25]. Potentially conflicting results regarding the concurrence of residential turnover and neighbourhood unemployment and individual health deserve further exploration in general and in regard to questions of urban insomnia in particular.

Summarising, neighbourhood unemployment and residential turnover might precondition a vast range of intermediate sleep-related stressors in urban residential contexts. For this reason, the study focus on these two neighbourhood exposures might serve as a suitable starting point for an epidemiological exploration into the development of chronic ‘neighbourhood insomnia’.

Further clarification is also needed when it comes to individual social characteristics influencing residents’ capabilities to deal with stressors inscribed in social neighbourhood characteristics. If individual social characteristics do not adequately buffer neighbourhood stressors, psychosocial stress might become chronic because of the inevitably chronic nature of residential neighbourhood exposures. Vulnerabilities for sleep complaints may occur in socio-economically disadvantaged individuals in terms of education and income, for example [26-29]: As an important determinant of health attitudes and (environmental) knowledge, education may influence sleep-related behaviour and sleep promoting strategies. Income may be specifically connected to psychosocial and physical sleep-related stressors stemming from material deprivation. Moreover, vulnerabilities may arise from social isolation which is a corollary of negative psychological states associated with sleep problems [30]. These individual social characteristics might additionally act as psychosocial stressors and modify urban neighbourhood stress effects on insomnia, eventually by way of disempowering residents’ capabilities to draw resources from their neighbourhoods.

**Figure 1:**
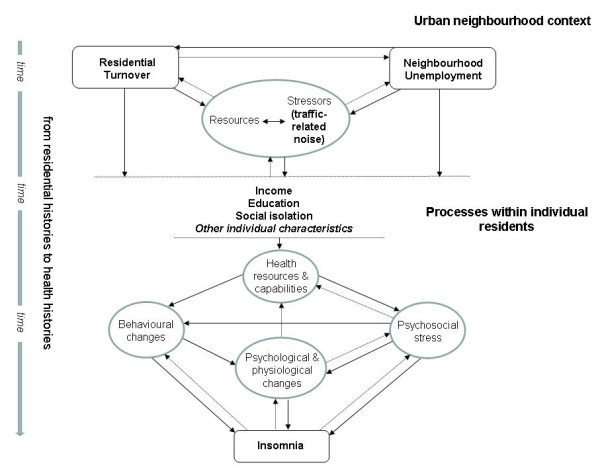
**Conceptual framework of insomnia as an outcome of multiple stressors in urban neighbourhood contexts.** In bold letters: sleep determinants operationalised by variables in this study; in slim letters: latent constructs; arrows: dependencies and pathways within the association of neighbourhood unemployment and residential turnover and insomnia; broken arrow lines: interrelations taking place over time. Individual social characteristics are placed between the neighbourhood context and individual processes in order to represent effect modification.

In these theoretical considerations, time is an inherent factor in the interplay of social-structural, intermediate and proximal stressors and resources across life-courses, since it takes time for social networks and ties to develop, social engagements and practices in urban neighbourhood collectives to grow, access to resources to be worked out, contextual stressors to be effective, and finally, psychological and physiological pathways to insomnia and health to evolve. From this time-related perspective, individual changes of residence may interfere with these complex mechanisms laid out here: If changes of residence disrupt neighbourhood relationships as well as the resources and stressors conveyed by them, residential ‘moves’ might be used to avoid contextual stressors in the current neighbourhood. Conversely, residents stuck in their place have longer durations of exposure to negative (or positive) neighbourhood stressors. Besides, they might be even more affected by their neighbourhoods, as neighbourhood unemployment and residential turnover could be particularly burdensome if there is no realistic escape from them. Up to now, insomnia studies concerned with social neighbourhood characteristics rarely consider exposure duration and change of residence during the observation period in their exposure assessments. Figure 1 summarises our study-specific conceptual framework of multiple stressors in relation to time guiding this study.

Against this background, we specify our research questions focussing on neighbourhood unemployment and residential turnover as major exposures as follows:

1. Are residents with chronic exposure to higher levels of neighbourhood unemployment and residential turnover more likely to report insomnia compared to those with lower exposure levels?

2. Are residents with relatively low income, low education, and social isolation especially vulnerable to higher levels of chronic neighbourhood exposures?

3. Does individual change of residence influence the associations between neighbourhood exposures and insomnia?

## Methods

### Study population and study sample

Individual data are derived from the Heinz Nixdorf Recall (HNR) Study, a population-based longitudinal study of 4814 randomly chosen participants (45–75 years of age) from three adjacent cities in the German Ruhr Area, Mülheim, Essen and Bochum [31,32]. Belonging to the former industrial core in Western Germany, these cities have been undergoing enormous economic restructuring [33,34]. Notwithstanding a large bundle of planning interventions in the last decades [35], the overall quality of life in the Ruhr Area still lags behind more prosperous, globalised regions in Germany, at least in terms of socio-economic measures. As a result, the general burden of chronic diseases and associated social risk factors are deemed comparatively high [36,37]. Additionally, processes of socio-spatial polarisation have been gaining momentum during the last ten years, rendering the study region all the more heterogeneous and suitable for epidemiological research concerned with social and health inequalities.

This study relies on individual level data gathered at the baseline medical examination of the HNR study (2000–2003). After giving written consent in accordance with the International Ethical Guidelines for Biomedical Research Involving Human Subjects (CIOMS) 1993, participants were interviewed face-to-face by trained personnel using computer-assisted questionnaires. Moreover, a sound medical examination including anamnestic interviews, blood samples, ECG, blood pressure measurement and many other examinations was conducted. The study was approved by the institutional review boards of the Ethical Commission of the Medical Faculty of the University of Duisburg-Essen and a quality management according to standard industrial norms (DIN EN ISO 9001: 2000) was implemented by TÜV Med Rheinland. Recruitment and potential sources of bias have been already evaluated and published elsewhere [38].

In order to build chronic exposure measures, participants’ residential histories were assessed retrospectively up to ten years before the baseline examination date using public registries administrated by the municipal authorities. Any changes of residence of people moving into, within or out of the respective city area are recorded in these registries. As no residential history data could be acquired for the city of Bochum, the sample had to be restricted to participants from the two neighbouring cities of Mülheim and Essen (N = 3033). We excluded participants with an incomplete history of residence to ensure a comparable exposure duration during the longitudinal observation period for all subjects (120 months, N = 2873, i.e. 94.7 per cent of the study population provided with information on residential history). Incompleteness of residential history mainly resulted from participants who had moved into the study cities after the beginning of the observation period or, vice versa, left the region during the period (N = 160). Except for a slightly younger age, a higher unemployment rate, and, naturally, more changes of residence, the population with incomplete residential data did not differ from the main sample.

### Measures

#### Outcome

In sleep epidemiology, there is still no universally applied standard definition for insomnia [3]. In this study, we used three insomnia symptoms, namely difficulties falling asleep (DFA), difficulties maintaining asleep (DMA), and early morning arousals (EMA). Participants were asked to report the weekly frequency of insomnia symptoms during the past four weeks before the examination using a standardised questionnaire [39]. This time frame already discloses persistent sleep problems and other chronic health conditions ( [40] for cardiovascular risks in the HNR study). Thus, this measurement of insomnia symptoms is appropriate for a study striving to assess the effect of long-term exposures. In our final outcome variable, we combined DFA, DMA, and EMA: If all three symptoms were present at least two times a week, participants were defined as insomniacs. Such a frequency-based cut-off point is consistent with previous studies on the epidemiology of insomnia [26,41].

#### Social neighbourhood characteristics (major exposures)

The addresses of residency during the observation period were linked to a predefined set of neighbourhood characteristics in order to estimate the individual exposure to area-based contextual stressors. Thus, addresses were not directly linked to the individual dataset to ensure data security. Neighbourhood data were retrieved from the municipal departments in charge of statistics and urban research in Mülheim and Essen and refers to 78 statistical units with an average of 11,747 residents. In part, these statistical units reflect grown structures and histories of former self-administered places. Other units have been created and reshaped for political reasons, aiming to make municipal administration and distribution of public means more efficient.

Two indicators were applied for the present analyses whose data availability and operationalisation should be generally accessible and replicable for standard small-scale area monitoring [42].

**Figure 2:**
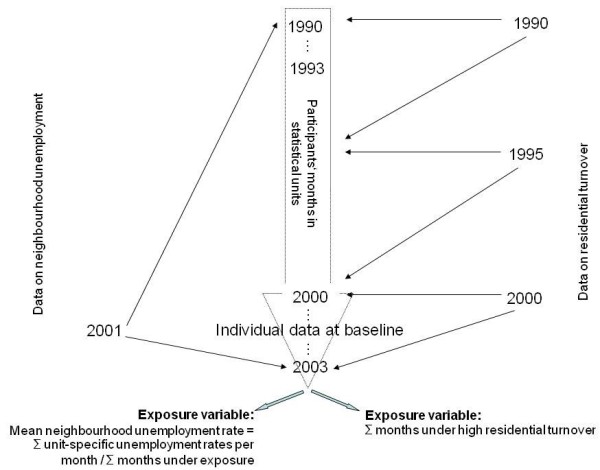
Data on social neighbourhood characteristics and their operationalisation as exposure variables.

First, considering neighbourhood unemployment as a chronic conditional frame for the evolution of sleep-related residential stressors (cf. Figure 1), we modelled this exposure as a neighbourhood status variable informing about mean unemployment rate throughout observation time. In general, neighbourhood unemployment rate is calculated as the percentage of unemployed residents related to the total economically active population. Monitoring of small-scale socio-spatial inequalities had not been introduced before the year 2000, which is why we had to use the rate values of the year 2001 for the past ten years. As exposure precedes outcome in our conceptual framework, participants examined in 2002 and 2003 were also given the rate values of 2001. In order to obtain the mean unemployment rate experienced by the participants in their residential history, we assigned the unit rates of the year 2001 to every participant’s address in each month registered by the residents’ registration authorities (Figure 2). After summing up the monthly values and dividing them by all months under observation, we categorised the continuous variable ‘mean neighbourhood unemployment rate’ into city-specific quartiles. In this way, we accounted for significant city-specific differences in the absolute level and range of neighbourhood unemployment, which might have distorted the relative meaning of unemployment in each city. The mean neighbourhood unemployment rates were 10.8% (range 13.9; IQR: 2.95) in Mülheim and 13.7% in Essen (range: 17.8; IQR: 5.62).

Second, given that social networks and resources are sought in the neighbourhood and are sensitive to the lengths of time periods under high residential turnover, we examined its effects on insomnia with a more time-dynamic exposure construct than the status variable neighbourhood unemployment. Residential turnover is operationalised as the sum of residents having moved into and out of the neighbourhood in relation to the total number of residents in the respective year. We acquired small-scale information from registry data for the years 1990, 1995 and 2000 identifying neighbourhoods with high residential turnover by the fifth quintile separately for Mülheim and Essen, which corresponds to 229–320 turnovers per 1000 residents in the 28 neighbourhoods of Mülheim and to 197–540 turnovers per 1000 residents in the 50 neighbourhoods of Essen (average across the three years of measurement). Data from 1990 was assigned to each month until December 1994, of 1995 until December 1999 and of 2000 until the last participants’ examination date in 2003 (Figure 2). Then we merged the residential turnover values with the individual data set and calculated the sum of months participants had lived in a neighbourhood with high residential turnover. Exposure classification was done by comparing participants never exposed to high residential turnover (N = 2218, 77.2%) with those affected at some time (N = 544, 18.9%) and with those exposed all the time (N = 111, 3.9%). This categorisation was applied because the bimodal distribution between the two extreme exposure categories did not allow for modelling exposure duration through a continuous variable.

#### Subgroups defined by individual social characteristics

In order to explore individual social vulnerabilities in the relationship between residential neighbourhood stress and sleep, we analysed subgroups of the study sample. Subgroups were defined using income and education as personal socioeconomic characteristics, as well as information on individual social networks. According to OECD recommendations, we computed the gender-specific equivalent income in our whole sample and classified participants as having relatively low income by the first quartile. Low education was indicated by less than 11 years of school and vocational education according to the International Standard Classification of Education 1997 (ISCED 97). Aside from socioeconomic disadvantages, social isolation as a third individual social characteristic was determined. In previous analyses on social inequality, social networks and social support in the HNR study population [43], the social integration index by Berkman et al. [44] has been found a reliable summary measure, encompassing partnership, close contacts regularly seen, and participation in social organisations. Participants in the lowest integration level of the index were termed socially isolated.

All three individual social characteristics were weakly, but significantly correlated.

#### Stratification by change of residence

Furthermore, analyses were stratified by change of residence. Participants who had been living at the same address for the whole observation period were classified as *non-movers* and those who had changed address at least once as *movers*. In comparing the estimates for these two contrasting groups, the impact of potential exposure discontinuities on the hypothesised neighbourhood associations should be figured out. For reasons of data security, we were confined to creating ‘exposure sum’ measures, only (see neighbourhood exposure measurement above). Therefore, it was not possible to rate whether a move was related to a significant change in neighbourhood exposures.

#### Background variables (covariates)

As background variables, we considered determinants which might not only have fostered the development of insomnia symptoms (e.g. [9,26,27,41,45]), but also influenced the individual participant’s location in neighbourhoods with their specific stressors [12,46,47] (cf. Figure 1):

We included years living under high exposure to traffic-related noise during the night as a physical stressor. Noise data is based on modelling according to the EU-Directive 2002/49/EG for the year 2006, mapped as isophone areas assigned to the participants’ addresses. We defined a relatively high exposure by a cut-off point of L_night, outside_ 60 dB (A), which exceeds the average night-time level of 40 dB (A) recommended by the Night Noise Guidelines of the World Health Organisation for Europe [48]. Above this threshold level, the prevalence of self-reported high sleep disturbances is estimated to be strikingly increased.

Next, we considered individual social characteristics, i.e. age (continuous), gender, and current economic activity (categorised as employed, economically inactive, retired, and unemployed). Following from age-related sleep changes and susceptibilities to sleep-disrupting chronic diseases, age is an important background characteristic for insomnia studies. Also, women are more prone to impaired sleep quality and insomnia than men. Economic activity structures daily activity patterns, sleep-wake-rhythms, time under neighbourhood exposure, and regular access to social resources other than those available in the neighbourhood. In the analyses for the whole sample, non-movers, and movers, we also adjusted for education as described above. In the low-income and socially isolated groups, we did not additionally account for education because the highly educated reference group (≥18 years) was completely missing among the insomniacs in both these subgroups.

### Statistical analyses

First, distributions of individual social characteristics, changes of residence, and social neighbourhood characteristics were compared between insomniacs and non-insomniacs for the whole sample and for the defined subgroups. Relationships between neighbourhood unemployment and residential turnover were explored both at the city and individual level by non-parametric correlations.

Apart from descriptive statistics, block-wise multivariate logistic regression analyses with insomnia as the dependent variable were conducted. Neighbourhood unemployment and residential turnover acted as major independent variables in separate regression models adjusted for relevant covariates. Since neighbourhood characteristics were operationalised as individual level means and months of exposure at the home address (allowing for changes of address), spatial clustering was not accounted for. However, multi-level modelling was applied for the subgroup of non-movers, yielding results no different from one-level logistic regression models. This is why main analyses were based on one-level multivariate logistic regressions, so that comparability of models between all the subgroups including movers was ensured.

In subgroup analyses, we employed the same statistical procedures in order to recognise modification of associations between social neighbourhood characteristics and insomnia by individual social characteristics.

Further, stratification was performed in order to compare the strength of associations between the neighbourhood contextual characteristics and insomnia in participants without and with change of residence which were hypothesised to increase or decrease vulnerability.

The main analyses were conducted in SPSS, versions 17 and 19, the multi-level modelling was done in Stata, version 11.2.

## Results

Nearly 12 per cent of the participants reported having insomnia at least two times a week (Table 1). Women were more likely to complain about insomnia than men. Among insomniacs the proportion having low income and education and living in social isolation were significantly higher than among non-insomniacs.

**Table 1:** Characteristics of the sample, absolute numbers and proportions in per cent, N (%)

	**Whole Sample**	**Insomniacs**	**Non-insomniacs**	**p (Chi**^**2**^**)**
	N = 2873	N = 342 (11.9%)	N = 2531 (88.1%)	
***Individual characteristics***				
**Female**	1426 (49.6%)	229 (67.0%)	1197 (47.3%)	0.000
**Mean age (SD)**	59.97 (SD 7.7)	60.32 (SD 7.5)	59.92 (SD 7.8)	
**Economic activity**				
**Employed**	1157 (40.3%)	113 (33.0%)	1044 (41.2%)	0.000
**Economically inactive**	379 (13.2%)	68 (19.9%)	311 (12.3%)	
**Retired**	1168 (40.7%)	133 (38.9%)	1035 (40.9%)	
**Unemployed**	169 (5.9%)	28 (8.2%)	141 (5.6%)	
**Education**				
**≥ 18 yrs.**	297 (10.3%)	18 (5.3%)	279 (11.0%)	0.000
**14-17 yrs.**	666 (23.2%)	46 (13.5%)	620 (24.5%)	
**11-13 yrs.**	1576 (54.9%)	198 (57.9%)	1378 (54.4%)	
**≤ 10 yrs.**	334 (11.6%)	80 (23.4%)	254 (10.0%)	
**Income**				
**Lowest (first quartile)**	735 (25.6%)	111 (32.5%)	624 (24.7%)	0.002
**Social isolation**				
**Social Integration Index Level I**	215 (7.5%)	47 (13.7%)	168 (6.6%)	0.000
**Change of residence**				
**Never**	2317 (80.6%)	275 (80.4%)	2042 (80.7%)	0.905
**Ever**	556 (19.4%)	67 (19.6%)	489 (19.3%)	
***Neighbourhood contextual characteristics***				
**Mean unemployment rate**				
**Lowest (first quartile)**	682 (23.7%)	60 (17.5%)	622 (24.6%)	0.004
**Mid-low (second quartile)**	738 (25.7%)	79 (23.1%)	659 (26.0%)	
**Mid-high (third quartile)**	779 (27.1%)	106 (31.0%)	673 (26.6%)	
**Highest (forth quartile)**	674 (23.5%)	97 (28.4%)	577 (22.8%)	
**High residential turnover**				
**Never**	2218 (77.2%)	251 (73.4%)	1967 (77.7%)	0.104
**At some time**	544 (18.9%)	72 (21.1%)	472 (18.6%)	
**All the time**	111 (3.9%)	19 (5.6%)	92 (3.6%)	
**Mean years having lived under high noise at night time (SD)**	0.49 (SD 2.05)	0.62 (SD 2.30)	0.47 (2.01)	0.184*

* t-test.

For instance, low education and social isolation were more than two times more prevalent among insomniacs than among non-insomniacs. Regarding changes of residence, no differences between the two groups of movers and non-movers could be observed. Yet, social neighbourhood characteristics of insomniacs and non-insomniacs differed markedly as insomniacs were more often exposed to high neighbourhood unemployment rates and high residential turnover. By trend, insomniacs had been living comparably longer under high exposure to traffic-related noise at night, too.

At the city level, nonparametric correlations between neighbourhood unemployment and residential turnover ranged from 0.39 in 1990 to 0.47 in 2000. Nonparametric correlations between the mean unemployment rate during the ten years prior to the baseline examination and months lived under high residential turnover were weak to moderate, but slightly elevated among the subgroups defined by individual social characteristics (Table 2).

**Table 2:** Relationship between mean neighbourhood unemployment rate and months under high residential turnover during observation time

	**Whole Sample, N = 2873**	**Low-income, N = 735**	**Less educated, N = 334**	**Socially isolated, N = 215**	**Non-movers, N = 2317**	**Movers, N = 556**
Neighbourhood unemployment rate * months under high residential turnover	0.273**	0.294**	0.364**	0.313**	0.274**	0.253**

Shown are nonparametric correlations, with two-sided levels of significance, p < 0.01, in the whole sample and in the subgroups.

In the whole sample, chances of reporting insomnia increased in the third and forth levels of neighbourhood unemployment, even after taking individual social and demographic factors as well as yearly exposure to night-time noise as covariates in multivariate regression models into account (Table 3).

**Table 3:** Insomnia depending on mean unemployment rate experienced in the neighbourhood

***Whole Sample***	***N = 2873***	***%***	***Model 1: crude***			***Model 2: adjusted***^***a)***^		
**Neighbourhood unemployment rate**			***OR***	***[−CI***	***+CI]***	***OR***	***[−CI***	***+CI]***
**Lowest (first quartile, Ref.)**	682	23.7	1			1		
**Mid-low (second quartile)**	738	25.7	1.24	[0.87	1.77]	1.15	[0.80	1.64]
**Mid-high (third quartile)**	779	27.1	1.63**	[1.17	2.28]	1.43*	[1.02	2.02]
**Highest (forth quartile)**	674	23.5	1.74**	[1.24	2.45]	1.42*	[1.00	2.03]
**Model fit (Cox & Snell R**^**2**^**/ Nagelkerke`s R**^**2**^**)**				0.005 /	0.009		0.033 /	0.064
***Subgroup: low-income***	***N = 735***	***%***	***Model 1: crude***			***Model 2: adjusted***^***b)***^		
**Neighbourhood unemployment rate**			***OR***	***[−CI***	***+CI]***	***OR***	***[−CI***	***+CI]***
**Lowest (first quartile, Ref.)**	133	18.1	1			1		
**Mid-low (second quartile)**	163	22.2	1.92	[0.88	4.21]	1.87	[0.85	4.14]
**Mid-high (third quartile)**	212	28.8	2.35*	[1.12	4.93]	2.30*	[1.09	4.85]
**Highest (forth quartile)**	227	30.9	3.04**	[1.48	6.26]	2.90**	[1.39	6.02]
**Model fit (Cox & Snell R**^**2**^**/ Nagelkerke`s R**^**2**^**)**				0.015 /	0.027		0.028 /	0.049
***Subgroup: less educated***	***N = 334***	***%***	***Model 1: crude***			***Model 2: adjusted***^***b)***^		
**Neighbourhood unemployment rate**			***OR***	***[−CI***	***+CI]***	***OR***	***[−CI***	***+CI]***
**Lowest (first quartile, Ref.)**	44	13.2	1			1		
**Mid-low (second quartile)**	74	22.2	3.21*	[1.01	10.22]	3.82*	[1.17	12.45]
**Mid-high (third quartile)**	100	29.9	3.70*	[1.21	11.32]	3.99*	[1.28	12.48]
**Highest (forth quartile)**	116	34.7	3.65*	[1.21	11.03]	3.51*	[1.15	10.76]
**Model fit (Cox & Snell R**^**2**^**/ Nagelkerke`s R**^**2**^**)**				0.022 /	0.033		0.049 /	0.073
***Subgroup: socially isolated***	***N = 215***	***%***	***Model 1: crude***			***Model 2: adjusted***^***b)***^		
**Neighbourhood unemployment rate**			***OR***	***[−CI***	***+CI]***	***OR***	***[−CI***	***+CI]***
**Lowest (first quartile, Ref.)**	40	18.6	1			1		
**Mid-low (second quartile)**	46	21.4	0.85	[0.23	3.19]	0.91	[0.24	3.49]
**Mid-high (third quartile)**	67	31.2	2.38	[0.80	7.06]	2.17	[0.71	6.62]
**Highest (forth quartile)**	62	28.8	3.33*	[1.14	9.79]	3.32*	[1.11	9.96]
**Model fit (Cox & Snell R**^**2**^**/ Nagelkerke`s R**^**2**^**)**				0.046 /	0.071		0.078 /	0.121
***Subgroup: non-movers***	***N = 2317***	***%***	***Model 1: crude***			***Model adjusted***^***a)***^		
**Neighbourhood unemployment rate**			***OR***	***[−CI***	***+CI]***	***OR***	***[−CI***	***+CI]***
**Lowest (first quartile, Ref.)**	568	24.5	1			1		
**Mid-low (second quartile)**	591	24.8	1.37	[0.92	2.03]	1.28	[0.86	1.91]
**Mid-high (third quartile)**	623	26.9	1.82**	[1.25	2.65]	1.64*	[1.12	2.40]
**Highest (forth quartile)**	535	23.1	1.81**	[1.23	2.66]	1.48	[0.99	2.20]
**Model fit (Cox & Snell R**^**2**^**/ Nagelkerke`s R**^**2**^**)**				0.006 /	0.011		0.029 /	0.056
***Subgroup: movers***	***N = 556***	***%***	***Model 1: crude***			***Model 2: adjusted***^***a)***^		
**Neighbourhood unemployment rate**			***OR***	***[−CI***	***+CI]***	***OR***	***[−CI***	***+CI]***
**Lowest (first quartile, Ref.)**	114	20.5	1			1		
**Mid-low (second quartile)**	147	26.4	0.82	[0.37	1.82]	0.69	[0.30	1.60]
**Mid-high (third quartile)**	156	28.1	1.01	[0.48	2.16]	0.79	[0.35	1.80]
**Highest (forth quartile)**	139	25.0	1.46	[0.70	3.05]	1.31	[0.59	2.94]
**Model fit (Cox & Snell R**^**2**^**/ Nagelkerke`s R**^**2**^**)**				0.005 /	0.009		0.078 /	0.149

Multivariate logistic regression models for the whole sample and for subgroups: Odds ratios are shown for having insomnia (reference group: non-insomniacs) with 95% CIs.

Model 1: crude exposure, without any adjustment. Model 2 a): adjusted for years under high exposure to night-time noise, age, gender, economic activity, and education. Model 2 b): adjusted for years under high exposure to night-time noise, age, gender, and economic activity. Levels of significance: * p-value < 0.05, ** p-value < 0.01.

Subgroup analyses further showed that the strength of associations between neighbourhood unemployment and insomnia was modified by participants’ individual social characteristics. Odds of reporting insomnia were enhanced in financially disadvantaged participants as compared to the whole sample. A tendency in the same direction was found among socially isolated participants. For less educated participants, a rather strong association between neighbourhood unemployment and insomnia appeared in all the upper three strata of neighbourhood unemployment. Notably, adjustment for covariates had only a minor effect on the strength of the odds ratios for neighbourhood unemployment rates.

Among non-movers, associations were generally more pronounced than among participants who had changed address at least one time during the ten-year observation period, suggesting a beneficial effect of residential mobility.

In the whole sample, months under high residential turnover tended to be related to a higher insomnia risk, although odds ratios were generally smaller than for the neighbourhood unemployment rate (Table 4).

**Table 4:** Insomnia depending on months under high residential turnover

***Whole Sample***	***N = 2873***	***%***	***Model 1: crude***			***Model 2: adjusted***^***a)***^		
**High residential turnover**			***OR***	***[−CI***	***+CI]***	***OR***	***[−CI***	***+CI]***
**Never (Ref.)**	2218	77.2	1			1		
**At some time**	544	18.9	1.20	[0.90	1.58]	1.11	[0.83	1.48]
**All the time**	111	3.9	1.62	[0.97	2.70]	1.33	[0.78	2.25]
**Model fit (Cox & Snell R**^**2**^**/ Nagelkerke`s R**^**2**^**)**				0.001 /	0.003		0.032 /	0.061
***Subgroup: low-income***	***N = 735***	***%***	***Model 1: crude***			***Model 2: adjusted***^***b)***^		
**High residential turnover**			***OR***	***[−CI***	***+CI]***	***OR***	***[−CI***	***+CI]***
**Never (Ref.)**	537	73.1	1			1		
**At some time**	159	21.6	1.19	[0.73	1.93]	1.14	[0.70	1.86]
**All the time**	39	5.3	1.82	[0.83	3.98]	1.55	[0.70	3.46]
**Model fit (Cox & Snell R**^**2**^**/ Nagelkerke`s R**^**2**^**)**				0.003 /	0.005		0.017 /	0.029
***Subgroup: less educated***	***N = 334***	***%***	***Model 1: crude***			***Model 2: adjusted***^***b)***^		
**High residential turnover**			***OR***	***[−CI***	***+CI]***	***OR***	***[−CI***	***+CI]***
**Never (Ref.)**	241	72.2	1			1		
**At some time**	69	20.6	1.45	[0.79	2.65]	1.33	[0.72	2.47]
**All the time**	24	7.2	1.46	[0.58	3.71]	1.36	[0.53	3.50]
**Model fit (Cox & Snell R**^**2**^**/ Nagelkerke`s R**^**2**^**)**				0.005 /	0.008		0.030 /	0.044
***Subgroup: socially isolated***	***N = 215***	***%***	***Model 1: crude***			***Model 2: adjusted***^***b)***^		
**High residential turnover**			***OR***	***[−CI***	***+CI]***	***OR***	***[−CI***	***+CI]***
**Never (Ref.)**	161	74.9	1			1		
**At some time**	36	16.7	1.61	[0.71	3.69]	1.62	[0.67	3.93]
**All the time**	18	8.4	†			†		
**Model fit (Cox&Snell R**^**2**^**/ Nagelkerke`s R**^**2**^**)**				0.012 /	0.019		0.049 /	0.076
***Subgroup: non-movers***	***N = 2317***	***%***	***Model 1: crude***			***Model 2: adjusted***^***a)***^		
**High residential turnover**			***OR***	***[−CI***	***+CI]***	***OR***	***[−CI***	***+CI]***
**Never (Ref.)**	1842	79.5	1			1		
**At some time**	381	16.4	1.06	[0.76	1.49]	1.00	[0.71	1.41]
**All the time**	94	4.1	1.83*	[1.07	3.12]	1.53	[0.88	2.66]
**Model fit (Cox & Snell R**^**2**^**/ Nagelkerke`s R**^**2**^**)**				0.002 /	0.004		0.027 /	0.052
***Subgroup: movers***	***N = 556***	***%***	***Model 1: crude***			***Model 2: adjusted***^***a)***^		
**High residential turnover**			***OR***	***[−CI***	***+CI]***	***OR***	***[−CI***	***+CI]***
**Never (Ref.)**	376	67.6	1			1		
**At some time**	163	29.3	1.59	[0.94	2.71]	1.45	[0.82	2.54]
**All the time**	17	3.1	Â§			Â§		
**Model fit (Cox & Snell R**^**2**^**/ Nagelkerke`s R**^**2**^**)**				0.006 /	0.012		0.077 /	0.147

Multivariate logistic regression models for the whole sample and for subgroups: Odds ratios are shown for having insomnia (reference group: non-insomniacs) with 95% CIs.

Model 1: crude exposure, without any adjustment. Model 2 a): adjusted for years under high exposure to night-time noise, age, gender, economic activity, and education, Model 2 b): adjusted for years under high exposure to night-time noise, age, gender, and economic activity. Level of significance: * p-value < 0.05. † too small numbers in this exposure category as to calculate reliable odds ratios; 6 out of 18 social isolated participants suffered from insomnia. Â§ too small numbers in this exposure category as to calculate reliable odds ratios; 1 out of 17 movers suffered from insomnia.

Subgroup analyses revealed that odds ratios in the low-income group, in participants with lower educational degrees and in socially isolated participants were quite similar to those odds estimated for the whole study sample.

Like in the previous analyses with neighbourhood unemployment rates, movers were less affected by this long-term neighbourhood exposure. Non-movers, in contrast, were more likely to report having insomnia when they had experienced high residential turnover in their neighbourhood for a longer period, but this association was diminished when individual factors and years under night-time noise were controlled for.

Throughout regression analyses, inclusion of long-term exposure to night-time noise did not contribute to explaining insomnia, leaving effect estimates of neighbourhood unemployment and residential turnover largely unaffected – with one exception: In less educated participants, insomnia odds tended to be increased by 11 per cent per year under high exposure to noise (results not shown). Observation numbers were too small to yield reliable results, though.

## Discussion

Based on an approach of multiple stressors, we investigated if social neighbourhood characteristics such as unemployment and residential turnover were associated with insomnia and whether these associations were modified by individual social characteristics and change of residence.

In the whole sample, we observed a statistically significant association between neighbourhood unemployment and insomnia. If neighbourhood unemployment encompasses multiple contextual stressors, this finding could hint at precarious situations in regard to psychosocial and other urban stressors influencing residents’ sleep quality in deprived neighbourhoods. Our study region is the Ruhr Area, a formerly highly industrialised area which has experienced massive deindustrialisation during the past decades. Long-term unemployment, financial strain and associated disorder and stigmatisation certainly affect both individuals and neighbourhood communities. Sleep is termed an adaptive behaviour [5,6] and as a consequence of an uneasy and stressful neighbourhood life, a physiological mal-adaption could have a sleep-disturbing outcome expressed by difficulties falling asleep, maintaining sleep and early morning arousals. Because of their structural persistence, contextual stressors are capable of directly inducing stress bringing on insomnia. This mechanism may be mediated and modified by psychosocial-cognitive factors – resources and stressors alike – relating to residents’ perception and appraisal of their neighbourhood situation and consecutive behavioural responses. A lack of mastery and control transmitted by the social neighbourhood context could be connected to stress-related health outcomes and risk behaviour and further deteriorate sleep quality, for example [13].

Despite its weakness, the association between months lived under high residential turnover and insomnia might still indicate the existence of neighbourhood influences which result in negative emotional states detrimental to sleep (e.g. [30] for the effect of positive affect and psychological well-being). In particular, social networks and ties could fail to balance problems which emerge from an unstable social neighbourhood context. In deprived areas where population decline might have led to social fragmentation tendencies, suicide, use of drugs, disorders due to alcohol use, and assaults have been identified as leading causes of mortality [24], which might give credit to the idea of residential stability being beneficial for health. We do not believe that selective migration had greatly exacerbated the effect of neighbourhood unemployment on insomnia, for correlations of residential turnover and neighbourhood unemployment were merely moderate. Regarding migration in our study cities in the Ruhr Area, though, another aspect should be taken into consideration: In some inner city areas, residential turnover is highly correlated with (younger) foreign residents and migrants who are much more affected by socioeconomic disadvantage and health risks than their German counterparts. Owing to our explorative study design tentatively showing a concurrence of higher neighbourhood unemployment and residential turnover levels, we do not presume to discard the *social isolation hypothesis* adopted by others [20,25], as mentioned in the theoretical background of this paper.

Characteristics of urban life are far too complex to be captured by neighbourhood unemployment and residential turnover alone. It is a limitation of our study that we did not know exposures inside home as well as other sleep-related environmental resources and stressors beside noise. Here, the overall null result of long-term exposure to night-time noise might, in part, result from an imprecise exposure estimation using isophone values instead of the spatially more resolved façade values and from exposure misclassification due to lack of knowledge about residential characteristics (noise protection windows, location of bedroom and living room), personal habits (time spent at home, opening of windows, etc.) and personal characteristics such as hearing loss. However, the tendency of insomniacs having endured noise for a longer cumulative time might still point to noise effects adding up to psychosocial stress associated with high neighbourhood unemployment and residential turnover.

Even if we cannot infer Ruhr Area-specific combinations of stressors from our crude contextual data basis and even if we cannot uncover specific biopsychosocial mechanisms from our analyses, it is plausible to partially ascribe insomnia as well as sleep-adverse behaviour and psychosomatic factors to neighbourhood-related living conditions.

Our approach of multiply effective stressors was further confirmed by our subgroup analyses, with each individual social characteristic suggesting a dimension of potential ‘stress’ on its own. We may assume that maintaining a high sleep quality while being exposed to an adverse urban neighbourhood context could be hindered by disadvantages in income, educational attainment and social isolation. Urban societies are shaped by market exchange and price regulations, regimes of civil rights and redistributional welfare, reciprocally social resources in social networks as well as their interdependencies. If individual social characteristics constitute a vulnerable position in relation to these realms, they consequently account for locally unequal exposures to stressors and resources, health capabilities, including psychosocial constructs like self-efficacy, attitudes towards health and collective lifestyles, and health outcomes (cf. [49-51], cf. also with a special focus on systemic health inequalities).

The pronounced association of neighbourhood unemployment with insomnia in participants with low income supports the assumption of individual social stressors reinforcing the effect of neighbourhood deprivation on sleep and health. In participants with lower educational degrees, inconsistent associations of social neighbourhood characteristics with insomnia might not necessarily imply the irrelevance of the urban residential neighbourhood context. For instance, in less deprived neighbourhoods low education could mean being hampered in accessing available resources to one’s own health benefit or sleep hygiene practices if social thresholds are perceived as too high. In face of high neighbourhood unemployment, low education could also add to the impression of one’s own powerlessness to positively influence the neighbourhood situation. In consideration of the noise effect estimates in this subgroup, institutional rules such as legal rights for environmental protection might be difficult to interpret and apply in order to encounter physical stressors at the place of residence actively and successfully, for example. Moreover, our results give evidence of a high vulnerability of socially isolated persons who could have serious troubles to bolster up enough economic, cultural and social resources to care for a healthy diet [52], to assert themselves in their daily (neighbourhood) life or to mobilise public attention to health damaging housing conditions. Helplessness and incapability to access adequate health resources might be reasonable intermediate psychosocial stressors in the relationship between neighbourhood unemployment and insomnia. At the same time, individual social characteristics did not seem to increase vulnerability to residential turnover. This finding raises the question whether multiple individual problems combined with neighbourhood unemployment outweighs potential effects of other socially unsettling processes engendered by residential turnover. Correlations between mean neighbourhood unemployment rates and months under residential turnover showed a slight tendency for an increased double neighbourhood exposure among the subgroups, however, which might have amplified the effects of higher neighbourhood unemployment levels on insomnia.

Furthermore, we studied change of residence as an effect modifier in the association of neighbourhood contextual characteristics with insomnia. Effect estimates for neighbourhood exposures gained strength in participants who had not changed residence (non-movers). By contrast, we could not detect any effects of neighbourhood contextual characteristics on insomnia in movers. First of all, this finding supports our hypothesis of time shaping the relationship between neighbourhood and health, i.e. that an enduring exposure is more likely to negatively impact sleep quality. Moreover, in participants familiar with changing their socio-spatial networks, relations and resources as a consequence of their own residential mobility, the neighbourhood context might be of less importance for well-being and health. What is more, this finding presses us to explore reasons, patterns and effects of change of residence in the future. In order to assess the ‘harmfulness’ of social segregation for residents’ social exclusion, the capabilities of the ‘moving’ households are for urban planners and sociologists decisive to know. In view of the relatively high percentage of employed movers (48.9% vs. 40.3%, results not shown in Table 1), changes of residence will partially have to do with economic activity, but the motives are hidden and could be economic pressures, health problems or simply changes in need and lifestyle specific to the stage in life-course. The data did not allow us to follow past socio-spatial residential mobilities, and we could not tell whether a change of residential address meant a change of neighbourhood and exposure. This is a crucial aspect, since the material conditions of place of residence might infringe on life chances in the long term, especially for those “not (yet) in a ‘clearly weak’ socioeconomic position” ( [53], p. 184). Yet, we do not assume drastic up- or downward changes in the participants’ socioeconomic status. Movers had merely a bit more experience with higher neighbourhood unemployment levels than non-movers (28.1% and 25.0% vs. 26.9% and 23.1% in the third and forth level, results not shown in Table 1). Maybe, age structure of the cohort accounts for this result, because residents’ socioeconomic life-courses are determined earlier in life than at the ages of 35–65. In relation to the mean age of the study population, our retrospective observation period of ten years time might be too short. Also, there was no association between individual relocations in the past and insomnia at present (Table 1). Most probably, migration patterns in our sample were not driven by insomnia, though we cannot tell if this was the case with other medical conditions and leave this subject for future studies. Researchers should bear in mind then that psychosocial stress ensuing from difficult life situations, insomnia, and chronic ill-health could be well related to migration (cf. [54] for the case of rural–urban migrants in China). However, studies on urban neighbourhood exposures, change of residence and insomnia are largely missing which makes it difficult to give a solid and evidence-based interpretation of the results.

At this point, we like to draw the reader’s attention to two major methodological facets, i.e. the usage of statistical units as proxies for neighbourhoods, residential history as well as the operationalisation and modelling of neighbourhood characteristics.

No doubt the statistical units we referred to as neighbourhoods will not necessarily correspond to the places involved in the social interactions and time-activity-patterns relevant for residents’ sleep- and health-related resources and capabilities. Heterogeneous in origin and boundaries, these statistical units will contain various constellations of stressors and resources. Consequently, effects of neighbourhood characteristics might be underestimated. However: “A neighbourhood need not be homogenous to affect the lives of its inhabitants. In fact, complete homogeneity within in area precludes the study of contextual effects altogether” ( [55] p. 112). In a cross-sectional neighbourhood study with HNR study data [36], effects of neighbourhood unemployment on coronary calcification did not alter when a smaller statistical unit was used.

Alongside the need to consider life-course trajectories, epidemiology has become interested in residential history approaches and modelling cumulative exposures [13,56]. We tried to capture this aspect by relying on a ten year exposure history based on administrative data. The public source of data acquisition proved to be quite trustworthy for two cities: Firstly, residential history data for the participants was almost complete, with an effective rate of 95.6 per cent (1680 out of 1757) and 99.0 per cent (1640 out of 1656) for participants from Mülheim and Essen respectively. Secondly, outcome and socioeconomic characteristics of the study sample with a complete residential history data did not substantially deviate from the whole study population including participants with incomplete residential information. Thirdly, the residential mobility is obviously typical for the age groups covered in our analysis. In a study dealing with social inequalities and residential choice in another city in Germany, Cologne, ca. 24 per cent of the participants of the same age had changed their home address in a ten years time ( [46], special analysis). So, the residential history data enabled us to consider exposure duration while investigating the potential effect of moves and to recognise possible social differences underlying individual changes of residence, e.g. in movers, non-movers, and in those participants with a disrupted residential history. This latter subsample excluded from our analysis turned out to be a specific group in the population which should be thought of when interpreting results from cross-sectional studies: Incompleteness seemed to signal rather dynamic life stages, as if these participants had not been settled yet, even after moving into the study region.

Modelling of long term neighbourhood exposures has become rather sophisticated, using multiple-year measures [57], indices of cumulative area-based socioeconomic environments [58], neighbourhood trajectories and hierarchical latent growth curve modelling [59], for instance. Lack of retrospective socioeconomic neighbourhood indicators prevented us from setting up a true time series and adopting such complex approaches. Yet, we managed to build a socioeconomic neighbourhood status variable overcoming exposure misclassifications owing to changes of residence. Correlations of neighbourhood unemployment and residential turnover as well as the expertise of the municipal departments in charge of statistics and urban research revealed relatively stable city structures throughout the 1990s, which compensates in part the flaw in socioeconomic data availability. Furthermore, we developed a dynamic measure of residential turnover in the neighbourhood, though a bimodal distribution of exposure to residential turnover and small observation numbers in monthly exposure strata thwarted differentiating exposure durations in more detail. In principle, our measure of residential turnover is rather unique: As far as we know, residential (in-)stability has been mostly analysed in cross-sectional studies and is frequently operationalised as the percentage of residents having been resident in a neighbourhood for a certain time span, e.g. for five years of time. Although implying a perspective into the past, this previously used measure does not allow for long-term analysis relying on several points in time. Such a summary measure is necessary to quantify the cumulative impact of social neighbourhood contextual characteristics. Our study would have demanded a larger sample, however, in order to scoop out the dynamic of this measurement approach.

From 2000 onwards, German cities have been growing more and more fragmented, but public socio-spatial monitoring has been much improved, partly as a reaction to this development of social segregation. Prospectively, downward spirals wearing away neighbourhood resources may become more and more traceable as selective population dynamics are developing at a quicker pace. For this reason, our study continues to assess neighbourhood contextual exposures during follow-up, creating a longitudinal design at both individual and neighbourhood level, testing more precise neighbourhood exposure accumulation measures, and exploring the relationship between neighbourhood unemployment and residential turnover in an enlarged database. In this way, we might be able to identify critical neighbourhood windows in the ongoing process of aging, because growing economic and physical inactivity in daily life are thought to enhance susceptibilities.

## Conclusions

Our findings hint at multiple stressors being effective in both the neighbourhood context and individual resident, possibly reflecting precarious life situations undermining residents’ sleep and health chances. Moreover, our results suggest a temporal dependency in the association between neighbourhood and insomnia.

This study does not justify any recommendations for professions engaged with urban public health at this point in time. A key may lie with long-lasting interventions serving both urban populations *as well as* subgroups defined by specific individual social characteristics throughout the city *and* in specific neighbourhoods (cf. [60]), though.

## Competing interests

The authors declare that they have no competing interests.

## Authors’ contributions

NR was responsible for the statistical analyses and drafting and writing the manuscript. ND and JSch supervised the statistical analyses and helped to interpret the results as well as to revise the manuscript. ND and BH coordinated neighbourhood contextual and noise data acquisition, NR, KF, and AV prepared and manipulated these data and designed contextual exposure measures. ND and BH were head of the residential history approach. ND and SW participated in conducting the HNR study. ND, JSch, BH, AS, and SW critically reviewed the manuscript. RE and JS are principal investigators of the HNR study. All authors read and approved the final manuscript.

## Pre-publication history

The pre-publication history for this paper can be accessed here:

http://www.biomedcentral.com/1471-2458/12/810/prepub
